# Supplementary material for: Interview with an avatar: Comparing online and virtual reality perspective taking for gender bias in STEM hiring decisions
Source: PLoS One. 2022 Jun 7;17(6):e0269430. doi: 10.1371/journal.pone.0269430 (PMC9173647; doi:10.1371/journal.pone.0269430)
Supplement: S1 Data — (DOCX) [file pone.0269430.s001.docx]

Supporting information

Data underlying the findings of the research has been made available via Mendeley Data. <https://data.mendeley.com/datasets/626n8889yb/1> doi: 10.17632/626n8889yb.1
